# Supplementary material for: Cell Membrane Fatty Acids and PIPs Modulate the Etiology of Pancreatic Cancer by Regulating AKT
Source: Nutrients. 2024 Dec 31;17(1):150. doi: 10.3390/nu17010150 (PMC11722924; doi:10.3390/nu17010150)
Supplement: Supplementary file 1 [file nutrients-17-00150-s001.zip › Supplementary Materials caption.pdf]

**Supplementary Materials:** The following supporting information can be downloaded at https://www.mdpi.com/article/10.3390/nu17010150/s1: Figure S1: Dietary PUFAs influence the progression of pancreatic neoplasm. EK mice were fed standard (SD), ω3 or ω6 enriched diets (n=5) for 9 months. **(A)** Representative H&E images of each diet group and number of lesions were counted high power field of 5 different fields of view and averaged. **(B)** Representative images of trichrome staining for each diet group and corresponding fibrosis score (5 different fields of view). **(C) Representative** images of PCNA and CK-19 double staining with quantitation of number of positive PCNA nuclei per high power field (5 different fields of view). **(D-F)** The pancreas from EK mice were evaluated by H&E and scored by two independent investigators (CT and GM) for total number of CPNs lesions **(D),** fibrosis **(E)** and proliferation **(F),** counted per high power field of 5 different fields of view and averaged. **(G)** Western blot images of EK mice pancreata on each diet probing for total and phosphorylated ERK and AKT proteins. Downstream regulators of the AKT pathway were also probed including total and phosphorylated Foxo3a and BAD proteins. β-actin used as a loading control. Asterisks in the graphs above a group define significance against the SD control: *p<0.05; **p<0.005; ***p<0.0005. Bars indicate significant differences between two groups. Results are expressed as mean ± standard deviation (SD).; Figure S2: Exogenous supplementation of PUFAs in vitro modulates the composition of plasma membrane in pancreatic cancer cells. **(A)** Pancreatic cancer cell line Panc-1 was incubated for 48h with increasing doses of LA in combination with a fixed dose of DHA (5 and 40μM) and cell viability determined by MTT assay. All the assays were performed in triplicate and averaged. **(B)** The table shows the percentage of reduction in the viability of the different combinations of the two fats. Pancreatic cancer cell line Panc-1 was incubated for 48h with increasing doses of DHA, and DHA incorporation to phospholipids was assessed by capillary gas chromatography-mass spectrometry. **(C)** DHA incorporation to Phosphatidylcholine (PC), **(D)** DHA incorporation to phosphatidylethanolamine (PE) and **(E)** DHA incorporation to triglycerides (TGs). **(F)** GC/MS chromatogram of PUFA standards displaying compound peaks sequence that separated chromatographically. The peak of DHA is clearly separated from the rest of the fatty acids by this method. Asterisks in the graphs above a group define significance against BSA control: *p<0.05; **p<0.005; ***p<0.0005. Bars indicate significant differences between two groups. Results are expressed as mean ± standard deviation of the mean (SD). Figure S3: Exogenous supplementation of PUFAs modify lipid profile. **(A-B)** Dietary lipids modify the lipid profile of mice tissues. The influence of diet on the lipid composition of the livers of animals fed standard diet, ω3-enriched diet and ω6-enriched diet was quantified by GC/MS. These results were used to select the main fatty acid of each type responsible for the physiological effect observed *in vivo* for use in the *in vitro* assays. **(C)** Molecular mechanism proposed. Table S1: Rodent diet composition; Table S2: Detailed rodent diet composition.
