# Supplementary material for: Cell Membrane Fatty Acids and PIPs Modulate the Etiology of Pancreatic Cancer by Regulating AKT
Source: Nutrients. 2024 Dec 31;17(1):150. doi: 10.3390/nu17010150 (PMC11722924; doi:10.3390/nu17010150)
Supplement: Supplementary file 1 [file nutrients-17-00150-s001.zip › Supplementary Table S1.pdf]

Rodent Diet With 45 kcal% Fat With Various Fat Sources

**Product #**	**D12451**		**D07101001**		**D07101002**	
	**Standard Diet**		**ω-6 PUFA**		**ω-3 PUFA**	
%	**gm**	**kcal**	**gm**	**kcal**	**gm**	**kcal**
Protein	23.7	20	23.6	20	23.7	20
Carbohydrate	41.4	35	41.4	35	41.4	35
Fat	23.6	45	23.6	45	23.6	45
Total		100		100		100
kcal/gm	4.73		4.72		4.73	
**Ingredient**	**gm**	**kcal**	**gm**	**kcal**	**gm**	**kcal**
Casein, 80 Mesh	200	800	200	800	200	800
L-Cystine	3	12	3	12	3	12
Corn Starch	72.8	291	72.8	291	72.8	291
Maltodextrin 10	100	400	100	400	100	400
Sucrose	172.8	691	172.8	691	172.8	691
Cellulose, BW200	50	0	50	0	50	0
Soybean Oil	25	225	13	117	13	117
Lard	177.5	1598	0	0	0	0
Safflower Oil	0	0	189.5	1706	0	0
Menhaden Oil (with 200 ppm	0	0	0	0	189.5	1706
Flaxseed oil	0	0	0	0	0	0
tBHQ	0	0	0.0379	0	0	0
Mineral Mix S10026	10	0	10	0	10	0
DiCalcium Phosphate	13	0	13	0	13	0
Calcium Carbonate	5.5	0	5.5	0	5.5	0
Potassium Citrate, 1 H2O	16.5	0	16.5	0	16.5	0
Vitamin Mix V10001	10	40	10	40	10	40
Choline Bitartrate	2	0	2	0	2	0
Cholesterol	0	0	0.76	0	0	0
FD&C Yellow Dye #5	0	0	0.05	0	0.025	0
FD&C Red Dye #40	0.05	0	0	0	0	0
FD&C Blue Dye #1	0	0	0	0	0.025	0
**Total**	**858.15**	**4057**	**858.9479**	**4057**	**858.15**	**4057**
n6	31.658		155.5		23.8	
n3	3.725		1.260		59.626	
n3:n6	0.118		0.008		2.503	
Menhaden oil contains 350-400 mg cholesterol per 100 g fat						
The diets have been matched for cholesterol and tBHQ.						

Research Diets, Inc, 10/4/07
