# Supplementary material for: Cell Membrane Fatty Acids and PIPs Modulate the Etiology of Pancreatic Cancer by Regulating AKT
Source: Nutrients. 2024 Dec 31;17(1):150. doi: 10.3390/nu17010150 (PMC11722924; doi:10.3390/nu17010150)
Supplement: Supplementary file 1 [file nutrients-17-00150-s001.zip › Supplementary Table S2.pdf]

Diet	Standard Chow	Safflower oil (ω6 enriched)	Menhaden Oil (ω3 enriched)
Reference	D12451	D07101001	D07101002
Lard	**177.5**		
Menhaden Oil, ARBP-F			**189.5**
Safflower Oil, USP		**189.5**	
Soybean Oil	**25**	**13**	**13**
Total	**202.5**	**202.5**	**202.5**
C2, Acetic	0.0	0.0	0.0
C4, Butyric	0.0	0.0	0.0
C6, Caproic	0.0	0.0	0.0
C8, Caprylic	0.0	0.0	0.0
C10, Capric	0.1	0.0	0.0
C12, Lauric	0.2	0.0	0.0
C14, Myristic	2.1	0.0	13.1
C14:1, Myristoleic, n-9	0.0	0.0	0.0
C15	0.1	0.0	0.9
C16, Palmitic	36.8	13.5	29.4
C16:1, Palmitoleic, n-9	2.5	0.0	18.5
C16:2, n-4	0.0	0.0	3.1
C16:3, n-9	0.0	0.0	2.9
C16:4, n-4	0.0	0.0	2.9
C17	0.7	0.0	0.7
C17:1	0.0	0.0	0.0
C18, Stearic	19.8	4.9	5.4
C18:1, Oleic, n-9	64.1	25.7	21.2
C18:1, Elaidic, Trans	0.0	0.0	0.0
C18:1n7C, Vaccenic	0.0	0.0	0.0
C18:2, Linoleic	56.2	**155.3**	10.3
C18:2, Trans	0.0	0.0	0.0
C18:3, Linolenic	4.2	1.2	3.8
C18:3, n-6	0.0	0.0	0.0
C18:3, Trans	0.0	0.0	0.0
C18:4, Stearidonic	0.0	0.0	5.9
C19, Nonadecanoic	0.0	0.0	0.0
C20, Arachidic	0.4	0.0	0.4
C20:1	1.2	0.0	2.9
C20:2	1.4	0.0	0.4
C20:3, n-6	0.2	0.0	0.8
C20:3, n-3	0.0	0.0	0.0
C20:4, Arachidonic, n-6	0.5	0.0	4.0
C20:4, n-3	0.0	0.0	0.0
C20:5, Eicosapentaenoic, n-3	0.0	0.0	26.9
C21, Heneicosanoic	0.0	0.0	0.0
C21:5, n-3	0.0	0.0	1.4
C22, Behenic	0.1	0.0	0.2
C22:1, Erucic	0.0	0.0	0.6
C22:4, Clupanodonic, n-6	0.0	0.0	0.4
C22:5, Docosapentaenoic, n-3	0.2	0.0	5.3
C22:6, Docosahexaenoic, n-3	0.0	0.0	19.4
C24, Lignoceric	0.0	0.0	1.2
C24:1	0.0	0.0	0.4
