# Supplementary figures and images for: Cell Membrane Fatty Acids and PIPs Modulate the Etiology of Pancreatic Cancer by Regulating AKT
Source: Nutrients. 2024 Dec 31;17(1):150. doi: 10.3390/nu17010150 (PMC11722924; doi:10.3390/nu17010150)

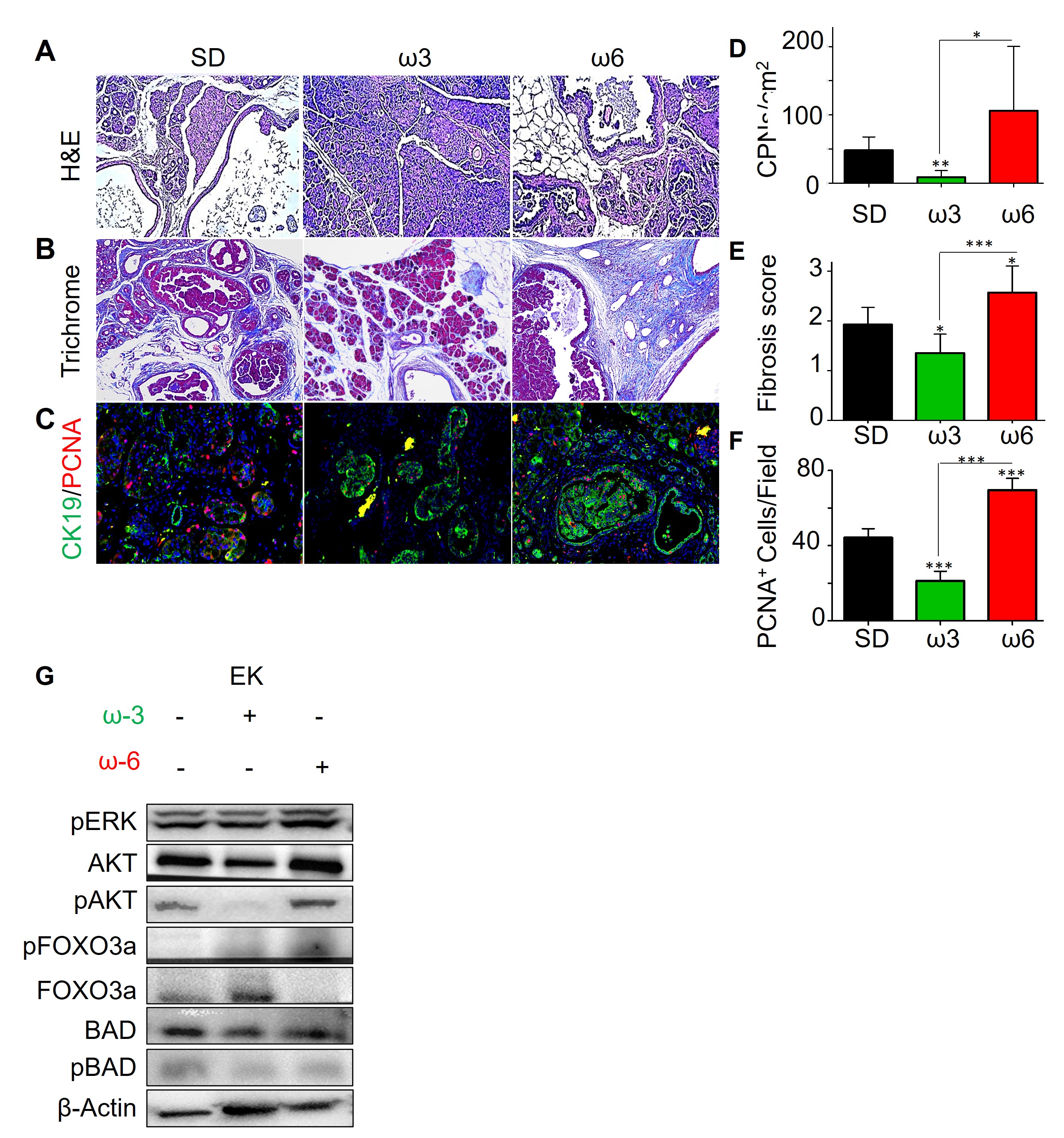

Supplement: Supplementary file 1 [file nutrients-17-00150-s001.zip › Supplementary Figure S1.jpg]

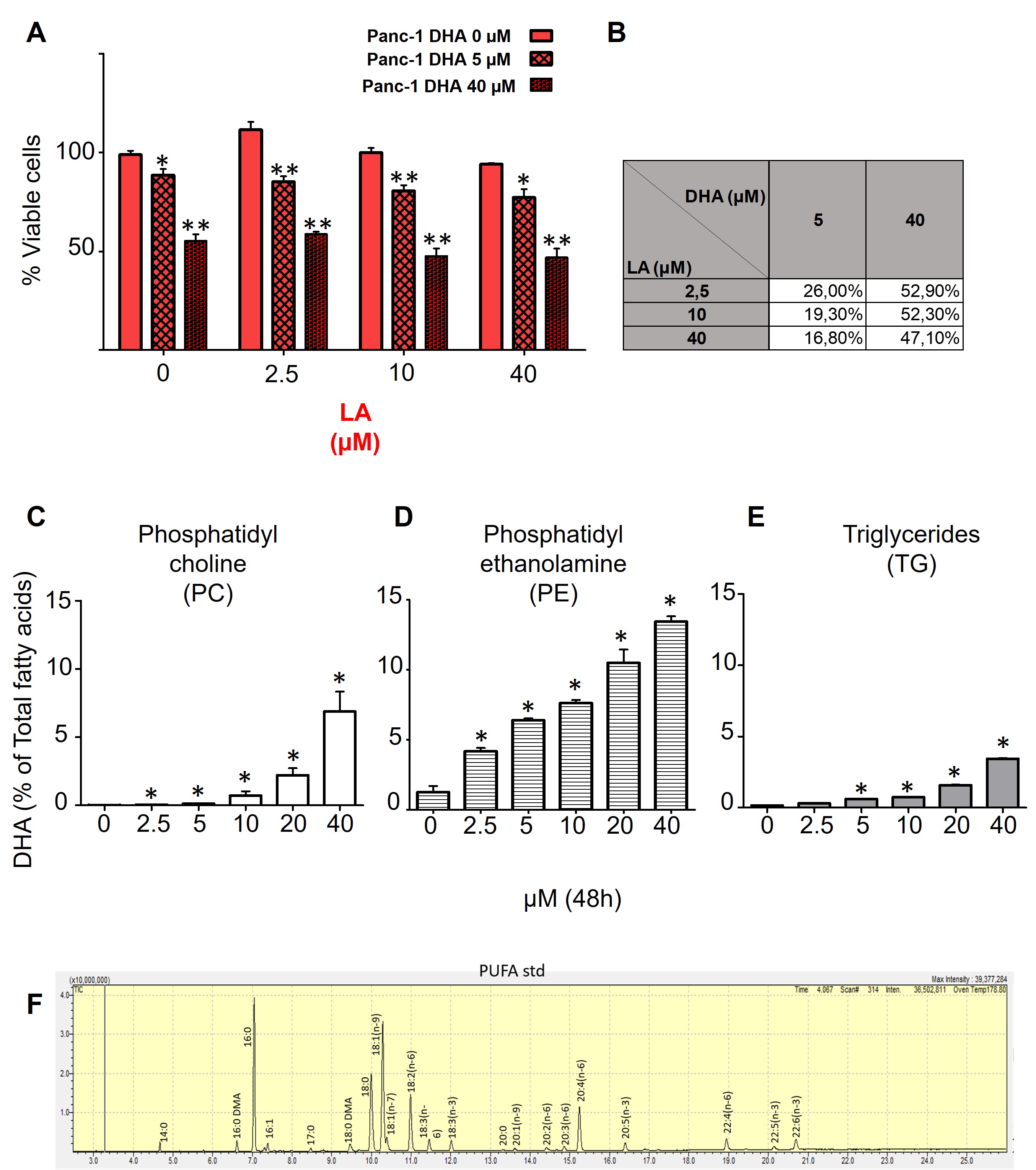

Supplement: Supplementary file 1 [file nutrients-17-00150-s001.zip › Supplementary Figure S2.jpg]

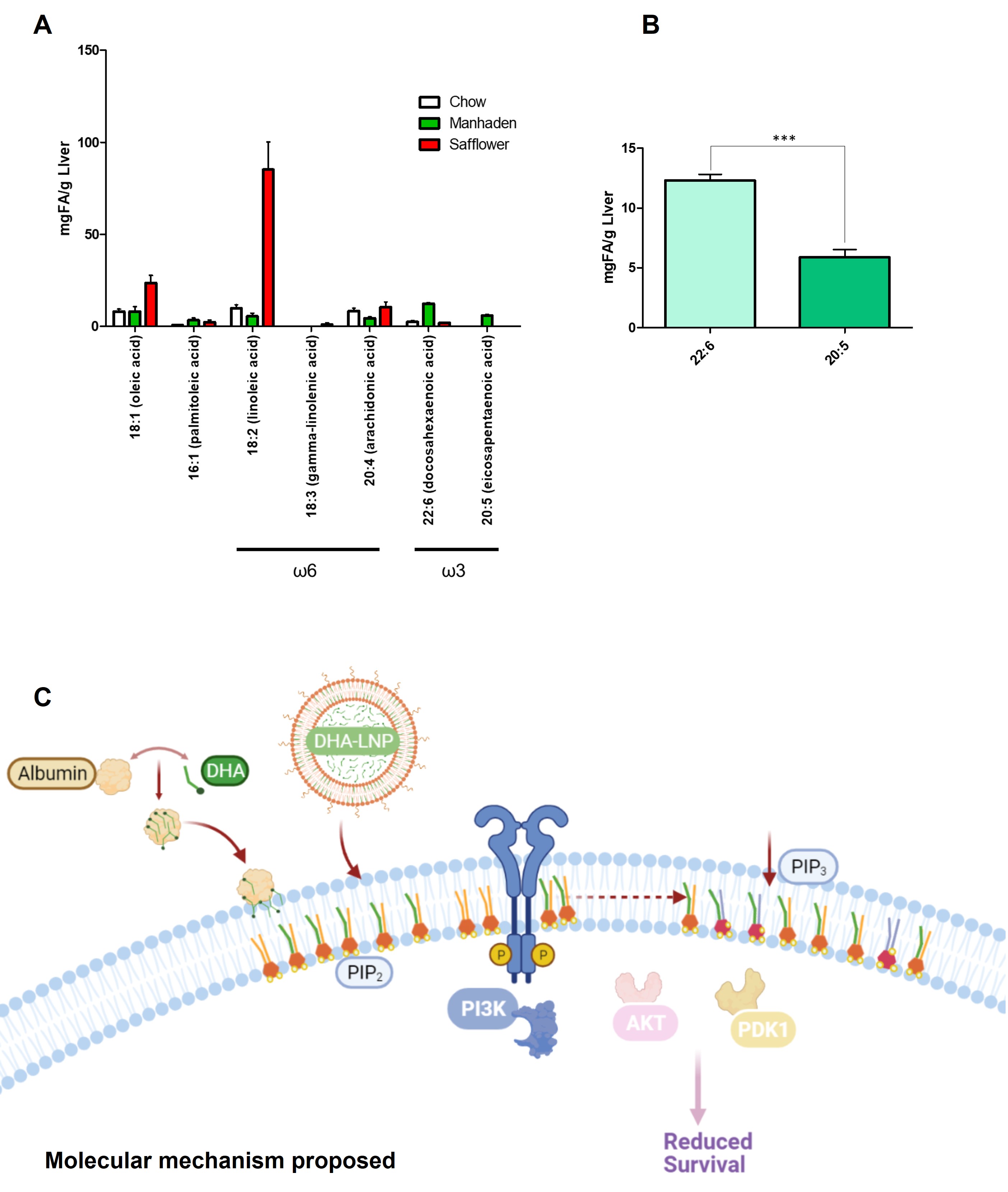

Supplement: Supplementary file 1 [file nutrients-17-00150-s001.zip › Supplementary Figure S3.jpg]
